# Interkinetic nuclear migration generates and opposes ventricular-zone crowding: insight into tissue mechanics

**DOI:** 10.3389/fncel.2014.00473

**Published:** 2015-01-28

**Authors:** Takaki Miyata, Mayumi Okamoto, Tomoyasu Shinoda, Ayano Kawaguchi

**Affiliations:** Anatomy and Cell Biology, Nagoya University Graduate School of MedicineNagoya, Aichi, Japan

**Keywords:** interkinetic nuclear migration, cortical development, time-lapse imaging, neural progenitor cells, cell division, slice culture, crowding, mechanical processes

## Abstract

The neuroepithelium (NE) or ventricular zone (VZ), from which multiple types of brain cells arise, is pseudostratified. In the NE/VZ, neural progenitor cells are elongated along the apicobasal axis, and their nuclei assume different apicobasal positions. These nuclei move in a cell cycle–dependent manner, i.e., apicalward during G2 phase and basalward during G1 phase, a process called interkinetic nuclear migration (INM). This review will summarize and discuss several topics: the nature of the INM exhibited by neural progenitor cells, the mechanical difficulties associated with INM in the developing cerebral cortex, the community-level mechanisms underlying collective and efficient INM, the impact on overall brain formation when NE/VZ is overcrowded due to loss of INM, and whether and how neural progenitor INM varies among mammalian species. These discussions will be based on recent findings obtained in live, three-dimensional specimens using quantitative and mechanical approaches. Experiments in which overcrowding was induced in mouse neocortical NE/VZ, as well as comparisons of neocortical INM between mice and ferrets, have revealed that the behavior of NE/VZ cells can be affected by cellular densification. A consideration of the physical aspects in the NE/VZ and the mechanical difficulties associated with high-degree pseudostratification (PS) is important for achieving a better understanding of neocortical development and evolution.

## Undifferentiated neural progenitor cells exhibit interkinetic nuclear migration

**Figure 1:**
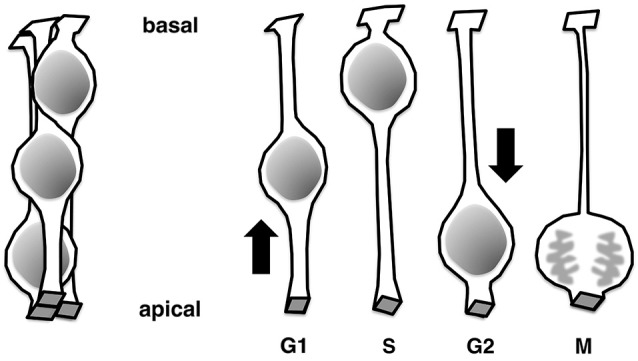
**Pseudostratification of epithelial cells achieved through interkinetic nuclear migration**.

The neural tube and walls of the early embryonic brain vesicles are composed entirely of undifferentiated progenitor cells (or “matrix cells” (Fujita, 1963)) and are referred to collectively as the neuroepithelium (NE) (reviewed in Götz and Huttner, 2005; Miyata, 2008; Taverna et al., 2014). Structurally, the NE is pseudostratified; that is, although there are several layers of nuclei in the NE wall (Figure 1, left), one layer of cells can host multiple layers of nuclei (Figure 1, right). Each cell extends to contact both the apical and basal surfaces of the wall, resulting in a bipolar cellular morphology with apical and basal processes. Nuclei of progenitor cells born at the apical surface of the NE move toward the basal side of the NE during G1 phase of the cell cycle. After completing S-phase in the basal portion of the NE, the nuclei return to the apical surface, where they undergo division as their parent cells did. Collectively, these processes are referred to as interkinetic nuclear migration, INM (or IKNM), (Schaper, 1897; Sauer, 1935; Sauer and Walker, 1959; Sidman et al., 1959; Fujita, 1962, reviewed in Taverna and Huttner, 2010; Kosodo, 2012; Reiner et al., 2012; Spear and Erickson, 2012; Lee and Norden, 2013).

As development proceeds, brain walls thicken and the bipolar-shaped progenitor cells grow in length (matrix cells (Fujita, 1963) or radial glial cells (reviewed in Rakic, 2003; Götz and Huttner, 2005; Lui et al., 2011; Taverna et al., 2014)). In these thickening brain walls, a large neuronal zone emerges along the outer pial surface, whereas the inner ventricular zone (VZ) consists mainly of progenitor cell somata. Together, NE cells and undifferentiated VZ cells are referred to as apical progenitors (based on their division at the apical surface, Götz and Huttner, 2005; Lui et al., 2011; Shitamukai and Matsuzaki, 2012; Taverna et al., 2014). Similar to the early NE, the VZ is pseudostratified due to the INM behaviors of progenitor cells. The thickness of the VZ is defined as the range of INM, leaving the outer neuronal territory free of progenitors’ nuclei. In the neocortical VZ, the basal region is dominated by nuclei of cells in S-phase and late G1-phase, whereas more apical parts are filled with nuclei of early G1-phase and G2-phase cells (Takahashi et al., 1994; Hayes and Nowakowski, 2000). To preserve normal tissue integrity, cells in the NE/VZ need to maintain an apically attached morphology with apical localization of the centrosome. INM is affected, due to abnormal delamination, when molecules involved in this process do not function, e.g., in the absence of Cdc42 (Cappello et al., 2006) or aPKCλ (Imai et al., 2006; Baye and Link, 2007), which control the adherens junction, or SAS-4 (Insolera et al., 2014), which regulates centriole biogenesis.

## INM-mediated pseudostratification in the developing neocortex is extensive and increases cell production per unit apical area

INM is observed in a wide variety of epithelia, including those of non-nervous system tissues or non-vertebrate animals (Sauer, 1936; Fujita, 1960; Grosse et al., 2011; Meyer et al., 2011; Rujano et al., 2013; Yamada et al., 2013). NE of the zebrafish retina and hindbrain (Leung et al., 2011; Lee and Norden, 2013) and the mouse retina (Baye and Link, 2007) do not exhibit clear intra-NE segregation of nuclei of cells at different cell-cycle phases (as seen in the mouse neocortex, with S-phase nuclei localized basally and non–S-phase nuclei localized apically). Instead, nuclei of all cell-cycle phases (except M-phase) intermingle and are seen all along the apicobasal axis of the epithelium. This “intermingling” (non-segregation) pattern also arises in the Drosophila wing disk (Meyer et al., 2011) and the mouse embryonic ureteric tube (Yamada et al., 2013). The difference between the neocortical NE/VZ and non-neocortical NE or non-NE pseudostratified epithelia could be explained by that the trajectory of INM (i.e., the extent of basalward nucleokinesis) differs depending on the subtype of progenitor cells. In the retina, Baye and Link (2007) found that the more basal the nucleus moves, the more likely it becomes that the next division will lead to production of neurons. Another intriguing possibility is that the collective INM pattern in the neocortical NE/VZ reflects physical conditions, such as tissue volume, cell number, and cellular traffic/flow in a given space. Histological comparisons in embryonic mice have revealed that the NE/VZ is thicker and more persistently maintained in the neocortex than in the brain stem (Miyata, 2007). From an evolutionary standpoint, it is noteworthy that the neocortical VZ is much thicker in human than in mouse (Zecević, 1993; Bayer and Altman, 2006). These size-related observations suggest that pseudostratification (PS) in the neocortical primordium is the most extensive (i.e., in the apicobasal range of INM), and that neocortical NE/VZ would therefore be a good model to study how physical or mechanical issues or parameters at the level of tissue or cell communities may affect neural progenitor behaviors.

**Figure 2:**
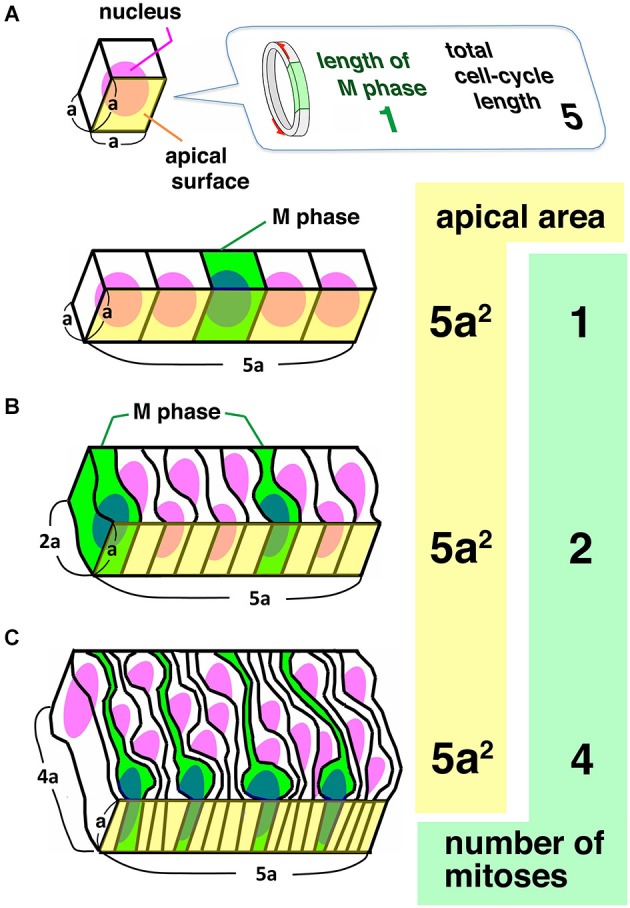
**Comparison between a simple cuboidal epithelium (A) and two columnar epithelia differing in the degree of pseudostratification (B, C)**. Given that there is a 1:5 ratio between the length of S-phase and the total length of the cell cycle length, and that there is no synchronization between neighboring cells, the number of mitoses at the unit of apical area increases as PS is accelerated, indicating that PS is a means to make epithelia as productive as possible at the apical surface.

What is the biological significance of high-degree INM-mediated PS of the type observed in the neocortical NE/VZ? In Figure 2, a simple cuboidal epithelium and two differently pseudostratified columnar (two-nuclei–and four-nuclei–deep) epithelia are compared. In the simple cuboidal epithelium, the length of each side of a cell is *a*. In the pseudostratified epithelia, the longer side of each apical endfoot remains *a*, whereas the other side of the apex shortens and the apicobasal length of each cell increases. The comparison reveals that increasing the degree of PS along the apicobasal axis may horizontally densify neural progenitors (i.e., increase the number of progenitors per unit of subapical volume and increase the number of mitoses per unit [5*a*^2^] of apical surface area). Therefore, high-degree PS allows an epithelial system to increase its productivity at the apical surface (Smart, 1972; Fish et al., 2008; Miyata, 2008).

**Figure 3:**
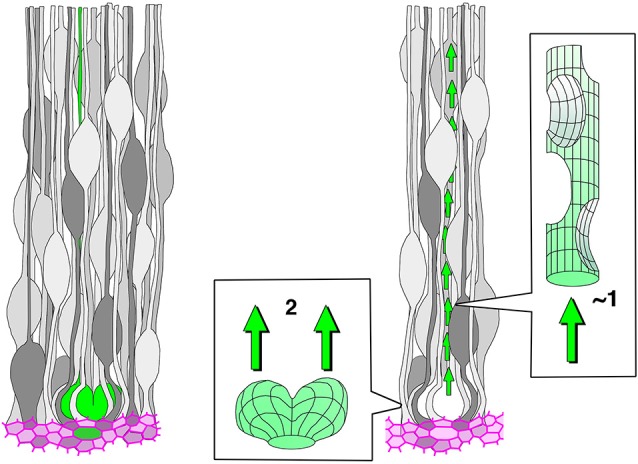
**A traffic bottleneck problem that presumably exists subapically in the ventricular zone (VZ) in the presence of high-degree pseudostratification (PS)**. The left panel shows a highly pseudostratified VZ; a horizontally dividing M-phase cell is colored in green, and the apical junction meshwork is colored in magenta. In the right panel, the space that the voluminous (expanding) M-phase cell inevitably occupies and the space through which its daughter cells must pass are compared. The latter space (outflow tract) may be much smaller due to VZ densification.

This discussion should be coupled with consideration of why NE/VZ progenitor cells divide at the apical surface. The centrosome is located in the apical endfoot due to the presence of a primary cilium (Paridaen et al., 2013; Insolera et al., 2014). Primary cilia are implicated in Wnt and Shh signaling as well as cell-cycle regulation (reviewed in Bisgrove and Yost, 2006; Fuccillo et al., 2006; Marshall and Nonaka, 2006), suggesting that progenitor cells need to possess an apical endfoot with a primary cilium in order to maintain their developmental potential and stem cell–like proliferation (Götz and Huttner, 2005; Cappello et al., 2006). Furthermore, the Delta–Notch interaction, which is important for the maintenance of stem-like cells, occurs at the apical surface (adherens junction) (Ohata et al., 2011; Hatakeyama et al., 2014). For undifferentiated NE/VZ cells connected to the apical surface, it seems beneficial to send the nucleus/soma to the apical endfoot in order to make the centrosome available for mitosis. Also, integration of newly generated daughter cells into the apical surface is easily achieved through apical mitoses. In the developing mouse neocortex, most apical mitoses occur with a cleavage furrow perpendicular to the apical surface, dividing each apical endfoot (Smart, 1973; Konno et al., 2008; Shitamukai and Matsuzaki, 2012; Figure 3). Consequently, daughter cells can easily and immediately join the apical meshwork. Thus, localizing mitoses to the apical surface is a favorable cytogenetic strategy for efficient expansion of undifferentiated NE/VZ cells in brain primordia. INM-mediated PS facilitates apical divisions, thereby supporting the maintenance/expansion of undifferentiated stem-like cells.

## What difficulties confront high-degree PS and collective INM?

Once we understand the aforementioned benefits of PS, we can then consider how it is efficiently achieved. In other words, we need to understand how INMs of NE/VZ cells are coordinated and assembled in an orderly manner. As an approach to addressing this question, it is useful to discuss several potential difficulties that NE/VZ cells need to overcome. Figure 3 illustrates, schematically but as faithfully as possible based on microscopic observations, cells in the VZ of the midembryonic mouse neocortex. All cells have an apical process that, together with the neighbor cells’ apices, constitutes the apical junction meshwork. An M-phase cell (green colored) undergoes cytokinesis horizontally (in an orientation parallel to the apical surface) to give rise to two daughter cells. The right panel highlights the relationship between initial volume of the pair-generated daughter cells and the space that is allowed as a route for the daughter cells’ nuclear migration. The outflow tract (a canal for basalward nucleokinesis by daughter cells) seems to be less than one-cell diameter due to the existence of other cells surrounding it at high density, while there are two nuclei (somata) to flow out. How can such a potential bottleneck problem be solved? Our time-lapse observations on slices prepared from H2B-mCherry transgenic mice (in which all nuclei are visualized) yielded a clear answer: pair-generated daughter cells usually move their nuclei basalward in a sequential, rather than simultaneous, manner (Okamoto et al., 2013). However, this observation raises a further question: how can daughter cells initiate basalward nucleokinesis sequentially?

## Basal process: a mother’s kind gift helps daughters’ traffic and brain formation

**Figure 4:**
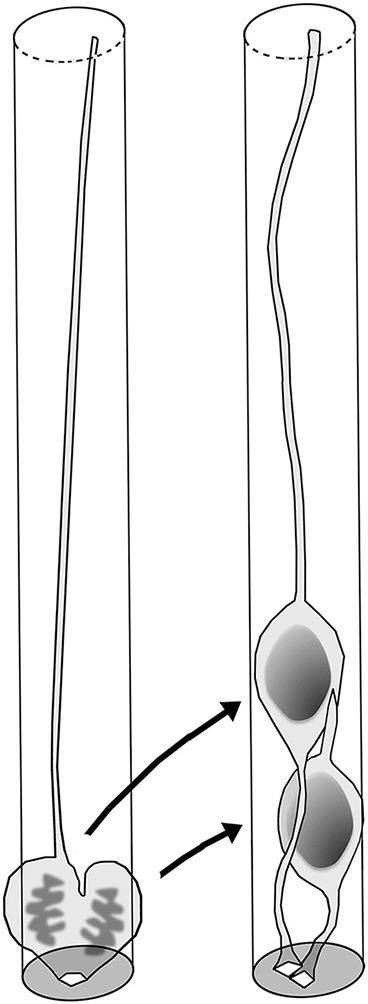
**Basal process–mediated sequential basalward nucleokinesis by pair-generated sister cells**. Each apically dividing M-phase cell’s basal process is maintained and inherited by one of its daughter cells (Miyata et al., 2001). The process-inheriting daughter cell exhibits quicker (more directional) basalward nuckeokinesis than its sister cell.

Time-lapse monitoring of daughter cells generated at the apical surface of VZ in slice culture was followed by quantitative analysis of nuclear movements. Measurement of mean-squared displacement (MSD) was used to determine the relationship between the morphology of daughter cells and the directionality of their initial nuclear movement. If the MSD for a tracked nucleus has a linear relationship with elapsed time (i.e., the MSD graph exhibits a linear pattern), the movement of the tested nucleus is considered to have random tendencies (i.e., non-directional and fluctuating motion). If the MSD graph instead exhibits a positive curvature, the movement is considered to be directional or persistent (Norden et al., 2009; Leung et al., 2011). The basal process of each apically dividing M-phase progenitor in the NE/VZ with a certain minimum thickness (>50 µm) is inherited by one of its daughters (Miyata et al., 2001; Noctor et al., 2001). In nuclear MSD profiles of daughter cells generated from a single progenitor, a more directional pattern was observed in the process-inheriting daughter cell than in its sister cell (Okamoto et al., 2013). This mechanism, in which the process-inheriting daughter cell moves its nucleus more quickly than its sister cell, is analogs to “priority boarding” in air travel or “staggered commuting” in metropolitan railway systems, and may contribute to the normally prompt stratification of nuclei/somata in each outflow tract (Figure 4).

**Figure 5:**
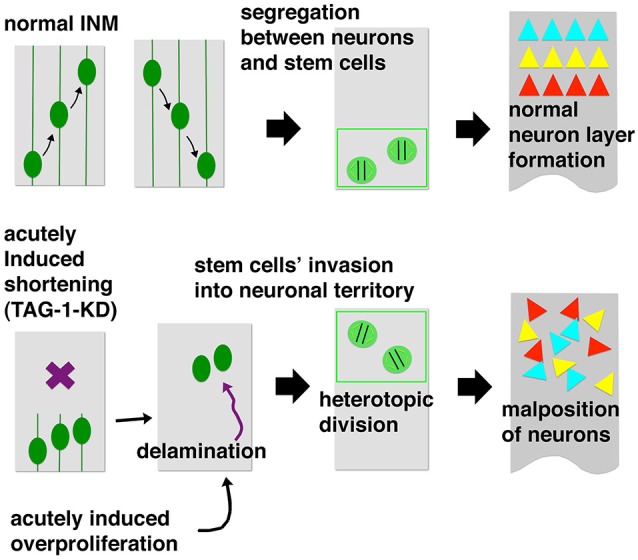
**Summary of acute TAG-1–knockdown (KD) experiments (Okamoto et al., 2013)**. TAG-1 shRNA was introduced through *in utero* electroporation at mouse embryonic day 10 (E10). By E12, VZ cells lost basal processes and were overcrowded near the apical surface. By E13, they detached from the apical surface and invaded the basal neuronal territory. The delaminated progenitors then heterotopically divided, generating different classes of neurons. Initial intermingling of neurons and progenitors (E13) and subsequent ectopic neuron production (~E17) disrupted neuronal layer formation, resulting in a mosaic-like abnormal distribution pattern.

If inherited basal processes are strictly analogs to priority boarding passes that streamline the flow of passengers under crowded conditions, the loss of these processes should lead to traffic problems such as severe overcrowding and congestion. Acute knockdown (KD) of the cell-surface molecule TAG-1 was used to determine the functional importance of the basal process and address the role of normal INM in overall brain formation (Okamoto et al., 2013). Upon loss of TAG-1, which is normally expressed in the basal part of mouse neocortical walls at embryonic day 10 (E10)–E11, VZ cells lost their basal processes by E12. Consequently, their basalward nucleokinesis was severely limited, causing their somata to accumulate near the apical surface (Figure 5, left part). By E13, these abnormally shortened VZ cells delaminated from the apical surface and invaded the basal area that should normally be occupied by neurons, thereby disrupting segregation of progenitor cells and neurons (Figure 5, center). The delaminated progenitors remained proliferative/undifferentiated (Pax6^+^) at heterotopic (far basal) positions until late in embryonic development (~E17). Nevertheless, distribution of neurons that were generated sequentially from these malpositioned progenitors was quite abnormal. Instead of forming layers, ectopically generated neurons were scattered almost throughout the wall in a randomized pattern (Figure 5, right part). This dysplasia therefore indicates that appropriate control of nucleokinesis within the early NE/VZ is important for preventing intermingling of progenitors and neurons, and thereby contributes to normal brain formation. However, why do the acutely shortened VZ cells in TAG-1–KD cerebral walls observed at E12 detach from the apical surface by E13?

## Experimentally induced acute overcrowding increases mechanical stress in VZ and induces abnormal delamination

**Figure 6:**
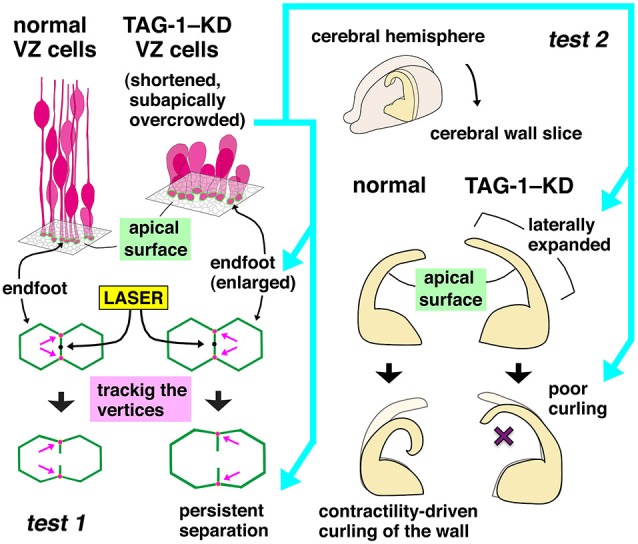
**Mechanical tests used for comparing normal and TAG-1–KD cerebral walls (Okamoto et al., 2013)**. In *test 1*, a pulse of UV laser was applied on the midpoint of a boundary line formed by two polygonal apices of VZ cells. Vertices at both ends of the laser-targeted side were tracked, and their separation was quantitated. The TAG-1–KD group exhibited greater and more persistent separations. In *test 2*, bending and curling of slices freshly prepared from normal or TAG-1–KD hemispheres were monitored under a phase-contrast microscope. TAG-1–KD slices that subapically contained many overcrowded (shortened) VZ cells were stiffer, exhibiting no or poorer bending/curling.

Monitoring at E12 revealed that the shortened TAG-1–KD VZ cells were overcrowded (subapically about 20% denser than in the normal VZ) (Figure 6, left top corner). Prompted by the hypothesis that VZ cells leave the apical surface when mechanical factors related to cell density increase to an intolerable level, reflecting high-degree proliferation (Smart, 1965, 1972), a series of experiments analyzed the physical condition of the overcrowded TAG-1–KD VZ. Microsurgical techniques such as laser ablation or making slices from hemispheric walls can be used to observe the mechanical conditions of cells or tissues of interest. If a certain portion is under tension or compression *in vivo*, the incision edges or freed tissue portions will then move according to the original mechanical conditions; these processes can be observed by microscopic monitoring. For example, laser ablation on the apical surface (as in *test 1*, Figure 6) results in centrifugal movement of the released vertices from the ablation point, revealing that the apical surface is contractile (as a result of the action of actomyosin-dependent mechanisms) and must therefore be under tension. Also, slicing cerebral hemispheric walls allows them to apically bend or curl (*test 2*, Figure 6). These techniques (destressing or stress-release tests) revealed that the subapical zone of the overcrowded TAG-1–KD VZ was indeed under excessive compression (as revealed in persistent separation of the tracked vertices in *test 1* and poorer bending/curling in *test 2*); this observation was further supported by *in silico* mechanical simulations (Okamoto et al., 2013). Thus, an overcrowding-induced delamination mechanism, such as the one recently reported in the *Drosophila* epithelium (Mariani et al., 2012), may also function in the developing mammalian neocortex. Progenitors evacuate (or are forced to exit) from the VZ in response to excessive acute mechanical stress.

Similar delamination of undifferentiated progenitors also occurred when another acute physical load on VZ cells was imposed by over-proliferation, induced by artificial expression of Wnt3a (Okamoto et al., 2013; Figure 5, left bottom corner). In this experiment, the nuclear horizontal packing density in the VZ increased (by about 6%), concomitant with a thickening of the VZ (by about 20%) and a nuclear densification along the apicobasal axis (by about 11%), and these VZ-densified Wnt3a-overexpressed cerebral walls bent or curled poorly. The molecular mechanisms by which VZ cells sense and respond to these acute mechanical loads should be investigated using mechanobiological approaches (Mammoto et al., 2012; Heisenberg and Bellaïche, 2013; Iskratsch et al., 2014). Wnt3a increases self-maintaining (non–neuron-producing) divisions during early cortical development through activation of β-catenin (Munji et al., 2011). Notably, telencephalic walls of transgenic mice expressing constitutively active β-catenin produce basal heterotopia of undifferentiated progenitors (Chenn and Walsh, 2002). Excessive FGF signaling also increased excessive basal mitosis (Inglis-Broadgate et al., 2005). Overproliferation induced in the VZ via artificial shortening of G1 phase of the cell cycle resulted in the expansion of non-VZ progenitors (Lange et al., 2009; Nonaka-Kinoshita et al., 2013). These previously reported heterotopic mitoses in rodent models may be better understood in light of progenitors’ responsiveness to mechanical stress in NE/VZ. Abnormal expansion of VZ has also been reported in mice lacking Apaf1 (Cecconi et al., 1998; Yoshida et al., 1998), Caspase 3 (Kuida et al., 1996), or Caspase 9 (Kuida et al., 1998) (reviewed in Kuan et al., 2000). A more recent study, however, reported that both Apaf1-deficient and Caspase 9-deficient mice did not show NE/VZ overgrowth (Nonomura et al., 2013). If the expansion and abnormal fragmentation of the apoptosis-inhibited neocortical VZ is reproducible as reported in the initial studies (Kuan et al., 2000), it might be another useful material by which we could ask how delamination is induced by VZ densification.

## Physiological thickening and densification of VZ during development

**Figure 7:**
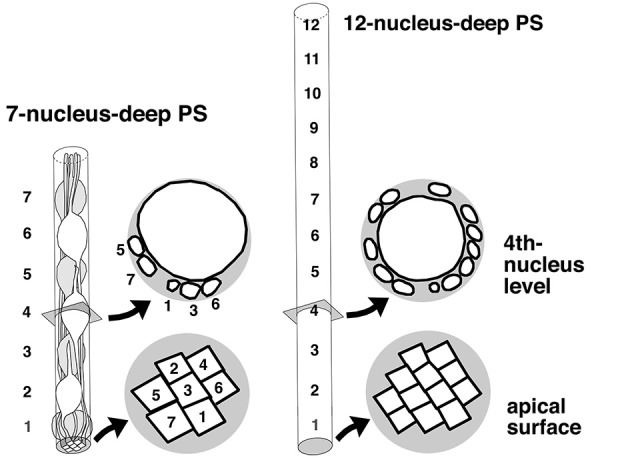
**Comparison of VZ cells’ spatial conditions between a moderate (7-nucleus-deep) and higher-degree (12-nucleus-deep) pseudostratification**. In the 12-nucleus-deep VZ, the short diameter of the nucleus/soma was reduced, and the apical endfeet may be more densified and individually reduced in size.

The observation of Wnt3a-induced thickening and horizontal cellular densification of the VZ provides a good opportunity for further discussion of whether (and, if so, how) the VZ thickening/densification that occurs physiologically during development and evolution might affect VZ cell behaviors. The thickness of the VZ is defined by the extent of PS along the apicobasal axis, i.e., by how many nuclei exhibiting INM are staggered from the apical surface toward the basal side (Sauer, 1935; Smart, 1972). It is likely that as more nuclei are stratified within the VZ, net apicobasal nuclear movements per unit of apical surface area tend to increase. In other words, as the VZ thickens, apicobasal nuclear traffic per unit volume of VZ becomes heavier. Figure 7 compares cell morphology between a VZ with 7-nucleus-deep PS and another with 12-nucleus-deep PS. The comparison is made within a cylinder-like hypothetical column, because time-lapse monitoring of H2B-mCherry-labeled nuclei showed that all nuclei move almost purely apicobasally, rather than horizontally (Okamoto et al., 2013). Probable differences between the two VZ columns with different degrees of PS include reduction in the short diameter of the nucleus/soma (due to the existence of other cells’ processes) and densification of the apical endfeet in the 12-nucleus-deep VZ.

During normal mouse embryonic development, the thickness of the neocortical VZ increases from E10 to E12 (Smart, 1973). Consistent with this, the density of apical endfeet also increases from E10 to E12 (Nishizawa et al., 2007). Notably, basal processes of apically dividing M-phase progenitors in a thin NE/VZ (e.g., E10 mouse cerebral walls or 24-hr zebrafish neural tubes) are often split or bifurcated (Kosodo et al., 2008), which does not occur in mouse cerebral walls at E12 and later. In early zebrafish neural tubes, split basal processes can be inherited symmetrically by two daughter cells (4/9 cases in Kosodo et al., 2008). These results raise the possibility that certain stage-dependent mechanisms that determine whether or not the basal processes are split and/or inherited symmetrically might involve mechanical stimuli, which are presumably weaker at E10 than at E12 when the VZ may be denser. However, asymmetric inheritance of basal processes, as observed in mouse cerebral walls at E12 and later, occurs widely in many different epithelia exhibiting PS: zebrafish neural tube and retina (Das et al., 2003; Kosodo et al., 2008; Alexandre et al., 2010), mouse and rat retina (Cayouette and Raff, 2003; Saito et al., 2003), mouse intestine (Grosse et al., 2011), and mouse ureteric bud (Packard et al., 2013). It is possible that asymmetric inheritance of the basal process contributes almost ubiquitously to management (spatiotemporal dispersion) of tissue stress generated through nuclear currents, and such management could be modified depending on the mechanical situation, which changes as development proceeds in a tissue-specific manner.

## Ferret–mouse differences in physiological VZ crowding and INM

The neocortical VZ is much thicker in human than in mouse (Zecević, 1993; Bayer and Altman, 2006). To investigate whether different mammalian species have evolved different strategies for cellular management of VZ nuclear traffic, a recent study compared mouse and ferret VZ at equivalent neocortical developmental stages (E13.5 in mice and E29–30 in ferrets), and found that intra-VZ cellular dynamics differ concomitantly with the thickening and densification of the VZ (Okamoto et al., 2014). Apicobasally, ferret VZ is thicker and slightly denser than mouse VZ: 16 stacks of nuclei in about 120-µm–thick ferret VZ (13.5 nuclei per 100 µm) vs. 12 stacks of nuclei in about 100-µm–thick mouse VZ (12.6 nuclei per 100 µm). Horizontally, the density of apical endfeet is greater (144%) (i.e., each apex is smaller) in ferrets than in mice. Also, horizontal nuclear density in the basal part of VZ is significantly higher in ferret (28 nuclei per 1000 µm^2^) than in mouse (22–24 nuclei per 1000 µm^2^). Nuclei are significantly more slender in ferret: the major axis (16.2 µm) is longer than in mouse (11.7 µm), and the minor axis is shorter (5.7 µm vs. 6.0 µm). These differences, obtained by imaging-based quantitation, are in line with expectations schematically shown in Figure 7.

**Figure 8:**
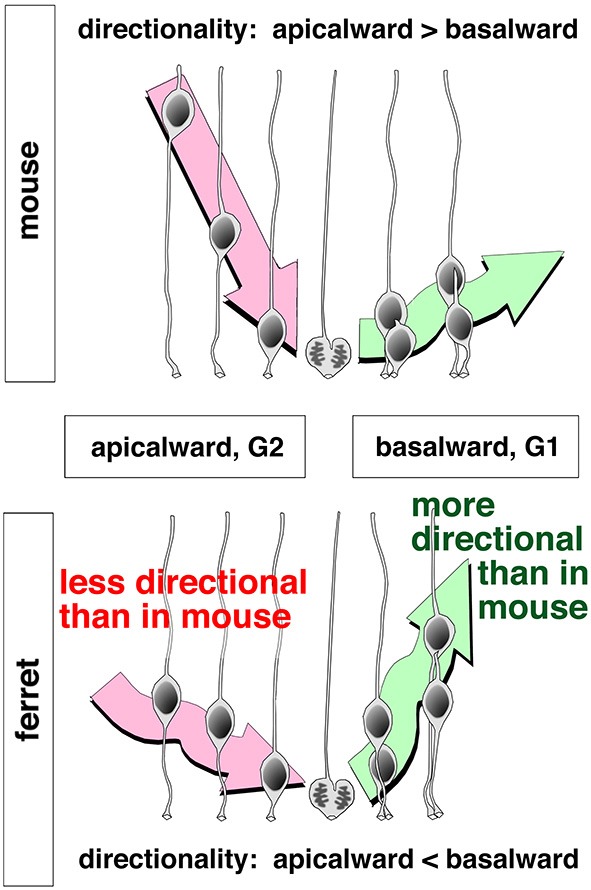
**Schematic illustrations depicting apicalward and basalward INM patterns and their differences between mice and ferrets (Okamoto et al., 2014)**.

In the mouse neocortical VZ, apicalward nuclear movements exhibited by G2-phase progenitors are highly directional (i.e., quick and persistent until they reach the apical surface), with non-linear MSD profiles (Okamoto et al., 2013), very similar to the directional apicalward nuckeokinesis observed in zebrafish retina and brain stem (Norden et al., 2009; Leung et al., 2011). By contrast, the basalward nucleokinesis exhibited by G1-phase mouse VZ cells is less directional (i.e., nuclei exhibited non-linear MSD profiles along the apicobasal axis), as is also the case for zebrafish cells (Norden et al., 2009; Leung et al., 2011). As mentioned earlier, initial basalward nucleokinesis is more directional in daughter cells that inherit the basal process (“BP”) than in daughter cells that do not (“nonBP”) (Okamoto et al., 2013; Figure 4). Accordingly, the directionality of nucleokinesis in the mouse neocortical VZ is ranked in the following order: apicalward > BP-basalward > nonBP-basalward (Figure 8, upper panel). Surprisingly, MSD analysis of ferrets revealed that directionality in the mid-embryonic ferret neocortical VZ is ranked in a different order: BP-basalward > nonBP-basalward > apicalward (Okamoto et al., 2014; Figure 8, lower panel). This finding suggests that although the basal process–mediated mechanism for differential initiation of nucleokinesis (Okamoto et al., 2013) is conserved between mice and ferrets, strategies for balancing flows to and from the apical surface differ between these species. Ferret–mouse comparisons at each phase of nucleokinesis suggested that the basalward phase is relatively accelerated, whereas the apicalward phase is decelerated, in ferrets. Future studies should investigate the molecular mechanisms underlying these differential nucleokinesis patterns between mice and ferrets. Whether physical conditions (such as elasticity or stiffness) vary between VZs of different nuclear density could be assessed quantitatively using atomic force microscopic (AFM) techniques (Iwashita et al., 2014).

## Conclusions and perspectives

**Figure 9:**
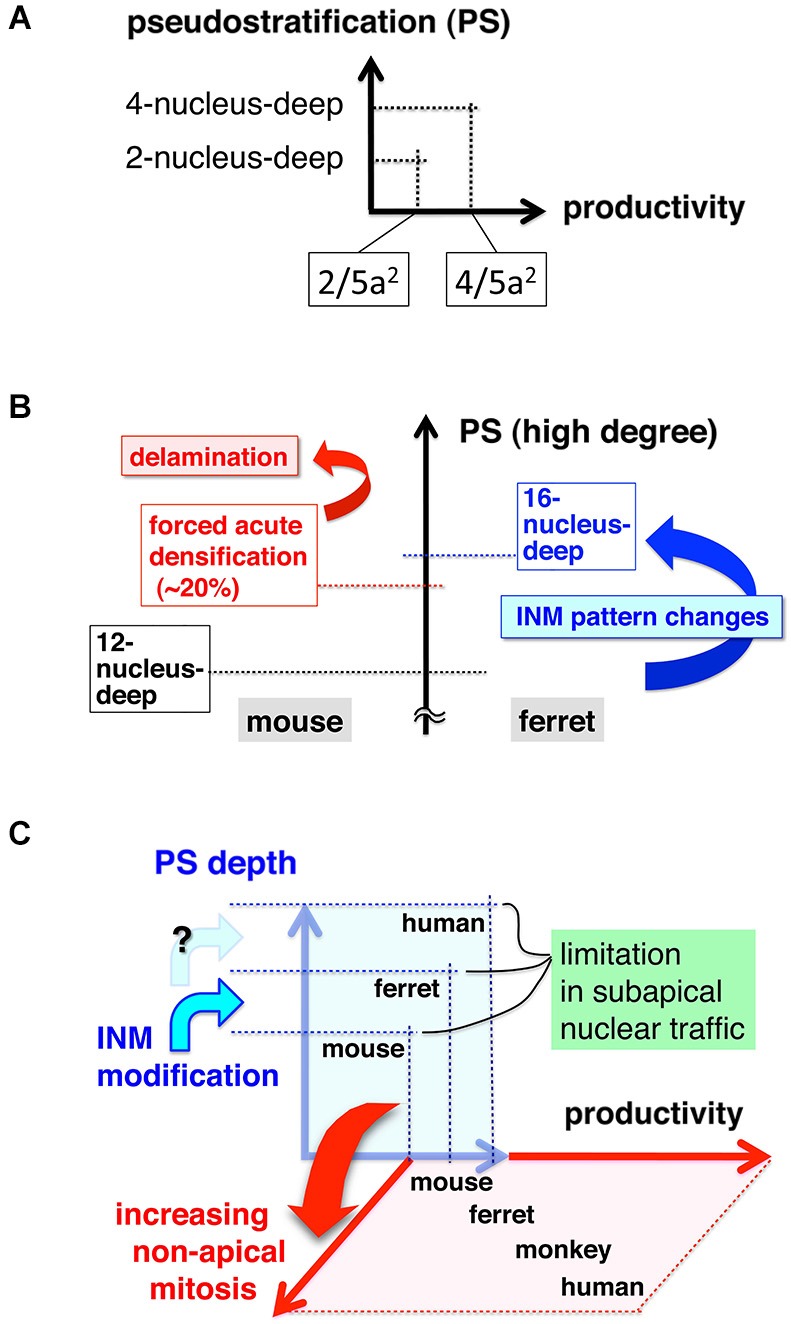
**Graphs depicting the relationship between the degree of pseudostratification (PS) and cell production at the apical surface (A, see also Figure 2), difficulties and INM modulation in high-degree PS (B), and a possible revolutionary change in the strategy for cell production from “PS-based apical” under physical/traffic limitations to “non-PS-mediated basal”, which is free from subapical traffic difficulties (C)**.

As discussed in the first part of this review, PS is an important means by which an epithelial system can increase its productivity at the apical surface (Figures 2, 9A). The second part of this review discussed the difficulties of high-degree PS from the viewpoint of nuclear traffic (Figures 5, 6). The apical surface is contractile and thus always spontaneously narrowing, although it receives M-phase somata that are expanding and voluminous (Figure 3). The co-occurrence of these two mechanically opposing phenomena is supported by the quick disappearance of newly generated G1-phase daughter cells’ nuclei from the apical surface. The exclusive use of the mother (M-phase) cell’s basal process by only one of its daughter cells facilitates the initial sequential (and thus non-competitive) nucleokinesis of the pair-generated sister cells’ nuclei away from the subapical space (the third part of this review, Figures 4, 8). This mechanism may collaborate with other mechanisms reported for basalward nucleokinesis during G1-phase: actomyosin-dependent (Schenk et al., 2009) and microtubule-dependent (Tsai et al., 2010) intracellular regulation, as well as passive basalward nuclear movements dependent on the apicalward nucleokinesis of other cells (Sauer, 1935; Norden et al., 2009; Kosodo et al., 2011; Leung et al., 2011).

The subapical space is physically limited, such that an acute 20% increase in the number of somata can result in abnormal delamination of undifferentiated cells (Okamoto et al., 2013; Figure 9B, left part). A similar increase in the load of nuclear traffic seems to be tolerable, if it occurs gradually during evolution. In the ferret VZ, which exhibits a higher level of PS than in the mouse VZ, the INM patterns (in both apicalward and basalward phases) are different from those in mice (Okamoto et al., 2014; Figure 8). This ferret–mouse difference suggests that modulation of INM may have allowed VZ cells to achieve high-degree PS, thereby increasing total cell production from the apical surface (Figure 9B, right part). However, such a thickened VZ would also encounter mechanical difficulties in the context of acute traffic problems, as shown experimentally in mice (Okamoto et al., 2013), probably limiting PS-based (apical) productivity. This discussion of traffic/mechanical difficulties in the PS system, based on recent live observations and experiments, prompted us to hypothesize that the mechanical conflicts may have caused the generation of new germinal layers during evolution (Figure 9C).

The idea that spatial limitations in the VZ may underlie the expansion of proliferative cells to the basal direction has previously been proposed based on histological observations using fixed specimens (Smart, 1965, 1972; Charvet and Striedter, 2011). Overproliferation induced in the VZ via artificial acceleration of the cell cycle resulted in the expansion of non-VZ progenitors (Lange et al., 2009; Nonaka-Kinoshita et al., 2013). Current research techniques allow us to quantitatively capture dynamic behaviors of cells, and to perform experimental manipulations that can change cells’ mechanical condition either indirectly or directly (reviewed in Mammoto et al., 2012; Heisenberg and Bellaïche, 2013). Therefore, future studies using mechanobiological approaches should be able to elucidate how a non-PS (non-VZ) proliferative zone for stem-like cells have arisen during neocortical evolution (Figure 9C).

In the research field of urban engineering, mass transportation is studied and developed in order to achieve better (i.e., safer, more economical, and more sustainable) quality of life and greater productivity. The human neocortical VZ is much thicker than the mouse neocortical VZ (Zecević, 1993; Bayer and Altman, 2006), giving us an impression that the former is more “urbanized.” We speculate that in such an extremely “urbanized” VZ, efforts to become as productive as possible at the apical surface would inevitably face increasing mechanical difficulties in subapical INM traffic. A shift from relying only on the PS to elaborating a new, non-PS cytogenetic method seems to present a mechanically reasonable solution to this challenge (Figure 9C). Recent studies have demonstrated that the outer subventricular zone (OSVZ), which contains undifferentiated progenitor cells (OSVZ [or basal] radial glia-like cells, oRG [bRG] cells), is a characteristic developmental feature of the human neocortex (Zecevic et al., 2005; Fietz et al., 2010; Hansen et al., 2010; Reillo et al., 2011; LaMonica et al., 2012; Lewitus et al., 2013). The OSVZ is also evident in non-human primates (Smart et al., 2002; Kelava et al., 2012; Betizeau et al., 2013) and ferrets (Fietz et al., 2010; Reillo et al., 2011; Martínez-Cerdeño et al., 2012; Reillo and Borrell, 2012; Poluch and Juliano, 2013). Although rodent neocortical primordia do not have cytoarchitechtonically distinct OSVZ-like structures, they have oRG-like progenitors (although they are much less abundant than in primates and ferrets) in regions basal to the VZ (Shitamukai et al., 2011; Wang et al., 2011; Martínez-Cerdeño et al., 2012; Tabata et al., 2012).

Vertical mitotic spindle orientation (leading to cytokinesis perpendicular to the apical surface) can contribute to the supply of non–apically connected VZ cells that move basally and eventually adopt an oRG-like morphology (Konno et al., 2008; Postiglione et al., 2011; Shitamukai et al., 2011; LaMonica et al., 2013); therefore, regulation of the cleavage orientation of stem-like cells at the apical surface may underlie the evolutionary changes that have generated the OSVZ. Recent studies on the mechanism regulating cleavage orientation have shown that the intracellular molecular machinery can be influenced by extrinsic factors such as diffusible or extracellular matrix proteins (reviewed in Théry and Bornens, 2006; Lancaster and Knoblich, 2012; Peyre and Morin, 2012; Shitamukai and Matsuzaki, 2012; Williams and Fuchs, 2013). In light of these results, future studies should attempt to determine whether cleavage orientation is regulated by tissue-level mechanical factors or through VZ densification.

Physiological delamination is exhibited by neocortical VZ cells that have acquired non–stem-like (differentiation) properties (Haubensak et al., 2004; Miyata et al., 2004; Noctor et al., 2004). This process can now be partly explained by a molecular mechanism similar to one that occurs during the epithelial-to-mesenchymal transition: downregulation of E-Cadherin by the Scratch transcription factors, which belong to the Snail superfamily (Itoh et al., 2013). Whether this process is also mechanically regulated, as speculated by Smart (1973), is another question that should be addressed experimentally. It is possible that spatial segregation of different classes of progenitors, which are also seen in non-neocortical NE/VZ tissues such as the developing retina (Weber et al., 2014), occurs under mechanical regulation. Direct mechanical manipulations have been useful for dissecting the molecular mechanisms underlying delamination in several model systems. For example, *Drosophila* and zebrafish embryos undergoing gastrulation have been manipulated using magnetic force (Brunet et al., 2013). In addition, the involvement of uterus-mediated external force in the specification of visceral endoderm cells in early mouse embryos was assessed by a culture system in which embryos were placed in chambers made with gels of different stiffness and by compressing embryos with an AFM cantilever (Hiramatsu et al., 2013). Application of such experimental methods, coupled with quantitative measurement of mechanical forces (as exemplified in this review, Figure 6), will deepen our understanding of both physiological (developmental and evolutionary) and pathological delamination (i.e., withdrawal from PS-based apical cytogenesis).

Finally, we are still far from understanding how INM behaviors of all VZ cells are coordinated such that they are not abnormally synchronized, in terms of both cell-cycle progression and nucleokinesis. One possibility worth investigating is that progression of the cell cycle is fine-tuned by cellular sensing of mechanical factors in the environment, and that such mechanosensation-based cell-cycle regulation might in turn regulate collective nucleokinesis. A combination of cell-biological experiments and *in silico* simulations should help to address this community-level question *in vivo*.

## Conflict of interest statement

The authors declare that the research was conducted in the absence of any commercial or financial relationships that could be construed as a potential conflict of interest.
